# Retraction: Selenium-enriched polysaccharides from *Pyracantha fortuneana* (Se-PFPs) inhibit the growth and invasive potential of ovarian cancer cells through inhibiting β-catenin signaling

**DOI:** 10.18632/oncotarget.28816

**Published:** 2025-12-31

**Authors:** Qianling Sun, Mengmeng Dong, Zhihui Wang, Changdong Wang, Deqiao Sheng, Zhihong Li, Debin Huang, Chengfu Yuan

**Affiliations:** ^1^College of Medical Science, China Three Gorges University, Yichang, HuBei 443002, China; ^2^Renhe Hospital of China Three Gorges University, Yichang, HuBei 443002, China; ^3^Molecular Medicine & Cancer Research Center, Chongqing Medical University, Chong qing 400016, China; ^4^Department of Pharmacology, Hubei Institute for Nationalities, Enshi, HuBei 445000, China; ^*^These authors have contributed equally to this work

**This article has been retracted:** This decision was made following an investigation by Oncotarget into concerns raised by readers and a subsequent request from the corresponding author. Our investigation identified multiple instances of internal and external duplications, overlaps, and reuse of Western Blot (WB) images. These include:

Internal Duplications:

Figure 1D: WB bands for PARP in HEY cells and SKOV3 cells overlap.

Figure 2B: WB bands for beta-actin in SKOV3 cells overlap with the beta-actin bands in HEY cells in Figure 5B.

Figure 3D: WB of beta-actin in SKOV3 cells was reused in Figures 6C and 6D, illustrating different treatments.

Figure 5A: WB bands for beta-catenin in SKOV3 cells overlap with HEY beta-catenin bands in Figure 5F.

Figure 5A: WB bands for Cyclin D1 in SKOV3 cells overlap with SKOV3 E-cadherin bands in Figure 6C.

Figure 5A: WB for Cyclin D1 in SKOV3 cells is the same as the HEY vimentin blot in Figure 6D.

Figure 5B: Beta-actin WB and Figure 5D beta-catenin WB in SKOV3 cells are the same.

Reuse of Images in a 2017 article [1]:

Figure 3D: Beta-actin image for SKOV3 cells was reused in Figure 5F [1].

Figure 5C: Beta-catenin image was found to overlap with the Caspase 1 image in Figure 4B [1].

Figure 5C: WB for GSK 3 beta was reused in Figure 5F as Caspase 1 WB [1].

Figure 6C: HEY cells’ beta-actin image is overlapping with the beta-actin image in Figure 4B [1].

Supplementary Figure 2B: WB bands of beta-catenin overlap with beta-actin bands in Figure 4B [1].

External Duplication:

The beta-actin bands in Figure 3D overlap with WB bands in Figure 4E in an unrelated paper [2].

Given these findings, the editorial decision has been made to retract the paper. Although the authors initially sent a retraction agreement letter, they did not confirm their agreement with the retraction text.

Original article: Oncotarget. 2016; 7:28369–28383. 28369-28383. https://doi.org/10.18632/oncotarget.8619
